# Addressing Venous Extravasation, a Concern in Intravenous Iron Administration

**DOI:** 10.7759/cureus.75323

**Published:** 2024-12-08

**Authors:** Catarina F Almeida, Soraia Carvalho, Alexandre Sarmento, Sílvia Amaral, Lúcia Borges

**Affiliations:** 1 Immunohemotherapy Department, Unidade de Saúde Local da Região de Aveiro, Aveiro, PRT

**Keywords:** anemia, endovenous iron, iron, skin pigmentation, venous extravasation

## Abstract

Intravenous iron is commonly used to treat anemia related to chronic illnesses, but venous extravasation, an uncommon side effect of intravenous iron, can result in persistent skin discoloration. This report presents the case of a female patient who experienced venous extravasation following intravenous iron administration, with data collected from her patient record. Although venous extravasation is a rare adverse effect, it is important for both patients and healthcare providers to recognize this potential complication. Adhering to established criteria to minimize the occurrence of extravasation and its associated risks is essential for improving patient outcomes.

## Introduction

Anemia is a frequent and significant complication in patients with inflammatory bowel diseases (IBDs), including Crohn's disease (CD), with a reported prevalence ranging from 6% to 74% depending on the patient population and disease severity [1,2]. Anemia in IBD patients is often multifactorial, with contributing factors such as chronic blood loss, malabsorption of nutrients, and the inflammatory process itself. The most common form of anemia in these patients is iron deficiency anemia, which results from a combination of chronic inflammation, decreased dietary intake, and poor iron absorption in the small intestine, often worsened by the disease’s effect on the gastrointestinal mucosa [3]. 

Oral iron supplementation is the first-line treatment for iron deficiency anemia in IBD patients. However, it is frequently poorly tolerated due to gastrointestinal side effects, including nausea, constipation, and abdominal discomfort [4]. Moreover, many patients with active disease experience inadequate absorption of oral iron due to the involvement of the small intestine in CD. In such cases, intravenous (IV) iron therapy has become an increasingly preferred alternative [4]. IV iron formulations, such as ferric carboxymaltose, have been shown to be highly effective in correcting iron deficiency anemia in IBD patients, with faster and more reliable outcome compared to oral preparations. IV iron therapy has the added benefit of bypassing gastrointestinal absorption, making it particularly useful in patients with active inflammation or previous bowel resections [5]. 

Despite its efficacy, IV iron administration carries the potential for adverse effects, with venous extravasation being a rare but notable complication. Venous extravasation occurs when an IV drug leaks out of the vein and into the surrounding tissue, leading to localized irritation, swelling, and sometimes tissue injury. While extravasation is more commonly associated with chemotherapy agents, it can occur with any IV infusion, including iron preparations. The risk of extravasation may be influenced by several factors, including the rate of infusion, the size of the vein, and the characteristics of the injected drug [6,7]. This complication, though uncommon, can lead to complications ranging from mild skin discoloration to severe tissue necrosis, emphasizing the importance of recognizing the signs early and taking preventive measures [7]. 

## Case presentation

A 93-year-old female patient, weighing 32 kg, with a history of stenosing CD of the small intestine and autoimmune interstitial pneumonia, was receiving corticosteroid therapy for her underlying conditions. Her past medical history was otherwise unremarkable. Due to poor response to oral iron supplementation, she was transitioned to IV iron therapy. The patient had previously tolerated IV iron without any complications. 

Pre-treatment laboratory results prior to administration of ferric carboxymaltose can be seen in Table 1.

**Table 1:** Laboratory results prior to the administration of ferric carboxymaltose. *Reference ranges according to the hospital's clinical pathology laboratory.

Parameter	Value	Reference range
Hemoglobin (g/dL)	10.9	11.5-16.5*
Mean Corpuscular Volume (fL)	88.3	76.0-96.0*
Mean Corpuscular Hemoglobin (fL)	29.1	27.0-32.0*
Ferritin (ng/mL)	93	22-322*
Transferrin Saturation Index (%)	13-16	20-50*
C-reactive Protein	0.23	0.00-0.50*

Based on these values, a dose of 1000 mg of ferric carboxymaltose (31 mg/kg) was prescribed, diluted in 250 mL saline, and infused at a rate of 600 mL/h. 

Due to the patient's age and venous fragility, venous access was difficult, and the infusion was ultimately administered into the cephalic vein of the left arm. No further IV therapies were given during this session. 

At the conclusion of the infusion, the patient developed localized skin swelling and a noticeable brown discoloration surrounding the site of the cannula. This was accompanied by mild edema and erythema, but there were no other signs of inflammation, such as warmth or tenderness. Importantly, the patient reported no discomfort or pain during or after the infusion. She remained hemodynamically stable, afebrile, and free of respiratory distress, pruritus, or any other significant symptoms. 

**Figure 1:**
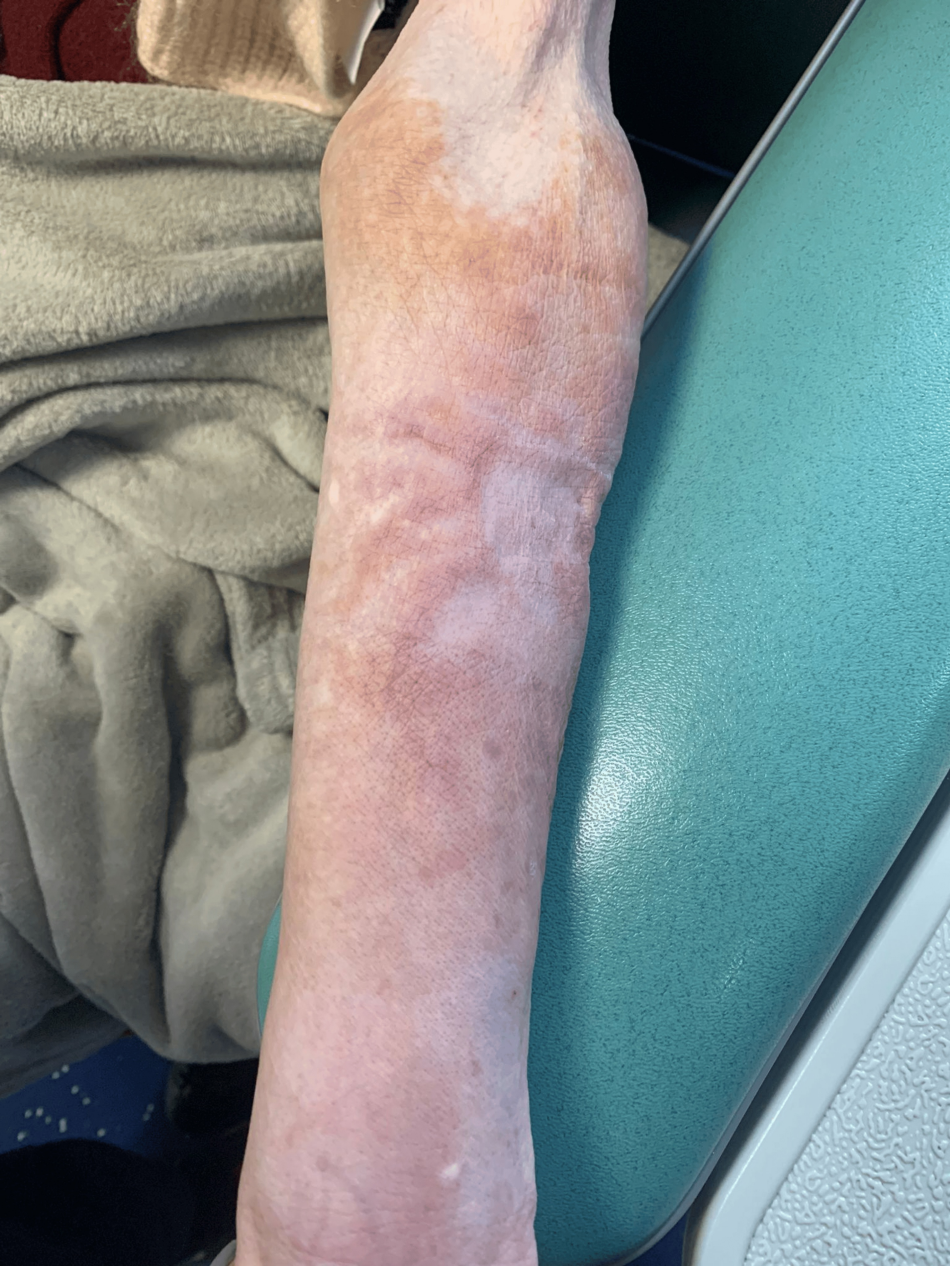
Skin staining on the day of the extravasation

On initial observation, the patient appeared well and had no complaints. Over the following days, the swelling in the left arm improved, while the skin pigmentation in the affected area progressively deepened, spreading across the entire anterior aspect of the arm. The discoloration remained localized and was not associated with any functional limitations, compression of underlying structures, or additional signs of inflammation (see Figure 1). 

**Figure 2:**
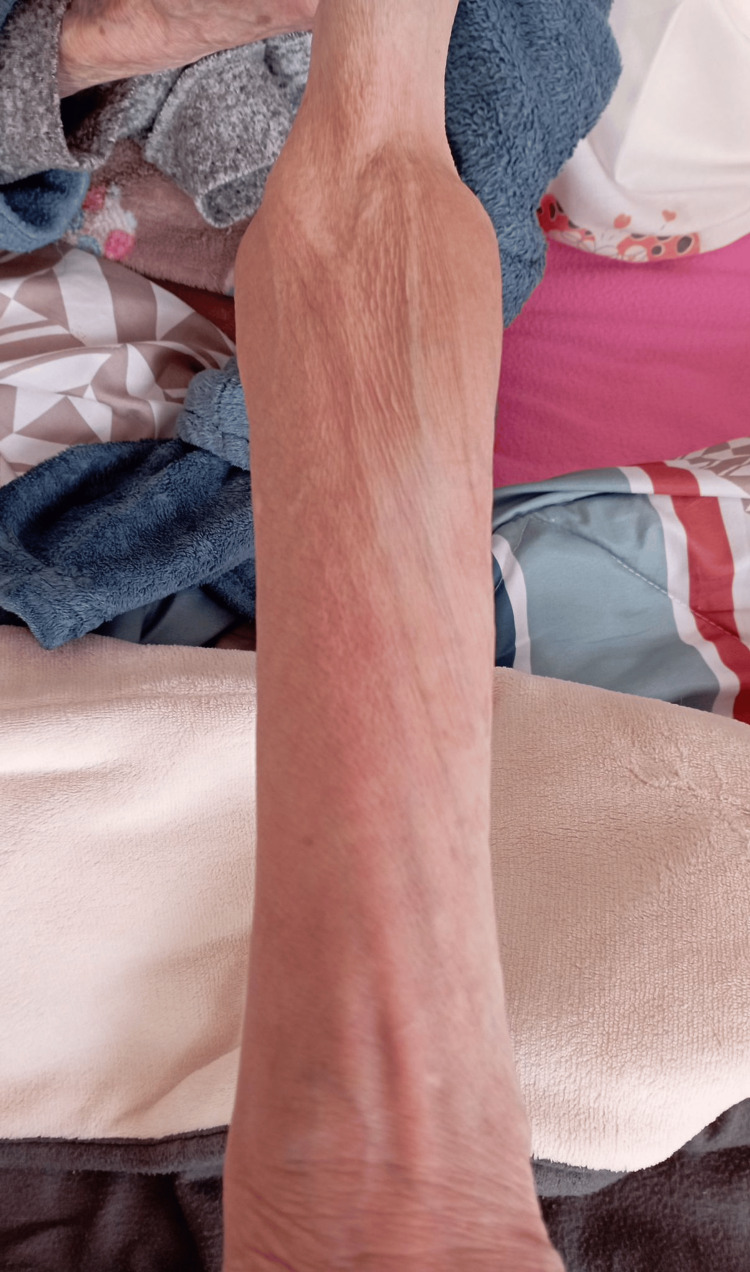
Skin staining one month after the day of the extravasation

At a follow-up visit one month later, the patient’s condition had improved. The cutaneous pigmentation had slightly faded along the borders, but it maintained its size and intensity. No signs of tissue necrosis or inflammatory changes were noted. The patient continued to be asymptomatic, with no further complications arising from the extravasation (see Figure 2). 

## Discussion

Venous extravasation occurs when the infused substance leaks out of the vein and into the surrounding soft tissue. Although extravasation can occur with many IV therapies, it is particularly notable with iron formulations, given the potential for significant local tissue injury and discoloration. Clinical manifestations of extravasation typically include pain, erythema, swelling, and, in some cases, blistering at the site of infusion. The severity of symptoms can vary depending on the volume of the extravasated substance, the duration of exposure, and the location of the infusion [7,8]. In our case, the patient experienced localized swelling and brown discoloration of the skin, which are common findings in extravasation of iron solutions. These symptoms may resolve over time, but in some cases, persistent pigmentation can remain for weeks or months, causing cosmetic concerns [8]. 

The risks of venous extravasation can be categorized into three broad groups: patient-related, procedure-related, and product-related factors. Patient-related factors include characteristics such as age, underlying vascular health, and the presence of comorbidities like obesity or dehydration, all of which can influence venous access and the risk of extravasation. Procedure-related factors refer to the technique of venous puncture and the rate of infusion. Difficult venous access, which was noted in our case, increases the likelihood of inadvertent extravasation, as does the rapid infusion of large volumes of fluid. Product-related factors concern the physical properties of the infused substance, such as osmolarity, viscosity, and pH. Iron formulations like ferric carboxymaltose can cause localized irritation and damage if extravasation occurs, due to their relatively high osmolarity and alkaline pH [6]. 

In preventing extravasation, several strategies are recommended. These include careful assessment of venous access prior to infusion, the use of appropriate veins with good blood flow, and ensuring that the infusion rate is controlled and appropriate for the specific iron formulation. Monitoring during the infusion process is crucial, particularly in high-risk patients, to detect any early signs of extravasation, such as swelling or pain at the infusion site. If extravasation is suspected, immediate measures such as discontinuing the infusion and applying cold compresses can help mitigate the local effects and prevent further tissue injury. In cases where the extravasation is significant, more advanced management may be required, including corticosteroid therapy or surgical intervention in rare instances of severe tissue damage [9]. 

## Conclusions

While venous extravasation is a rare event, healthcare providers should be aware of this complication when administering IV iron, particularly in older patients or those with fragile veins. It is essential to follow established protocols for IV infusion to minimize the risks and ensure the safe administration of IV iron therapy.

In our case, the patient did not experience any long-term adverse effects from the extravasation, and the pigmentation gradually faded over time, demonstrating that most cases of extravasation are self-limiting and resolve with conservative management. Nevertheless, its impact on esthetics, particularly in younger patients, cannot be overlooked, as it may lead to both physical and psychological consequences. Understanding and addressing the complexities associated with such occurrences is crucial to improving patient outcomes and ensuring a higher standard of care. 
